# Imaging of Ocular Surface Lesions Using Anterior Segment Optical Coherence Tomography

**DOI:** 10.1111/ceo.70081

**Published:** 2026-02-18

**Authors:** Wendy J. Li, Pragat J. Muthu, Anat Galor, Carol L. Karp

**Affiliations:** 1 Department of Ophthalmology, Bascom Palmer Eye Institute, Miami, Florida, USA; 2 Department of Ophthalmology, Miami Veterans Hospital, Miami, Florida, USA

**Keywords:** anterior segment optical coherence tomography, conjunctival lymphoma, conjunctival melanoma, ocular surface lesions, ocular surface squamous neoplasia

## Abstract

Ocular surface lesions encompass a wide spectrum of entities ranging from benign degenerative lesions to non-invasive and invasive malignant tumours. Accurate clinical differentiation is essential to ensure timely management. In this manuscript, we describe a series of benign and malignant ocular surface lesions and their clinical and specific imaging findings on anterior segment optical coherence tomography (AS-OCT), highlighting the diagnostic utility of this non-invasive imaging modality. We characterise ocular surface squamous neoplasia, pterygia, Salzmann’s nodular degeneration, conjunctival melanoma, primary acquired melanosis, conjunctival nevus, conjunctival lymphoma, benign reactive lymphoid hyperplasia and conjunctival amyloidosis. This series underscores the growing role of AS-OCT in supporting clinical assessment, guiding biopsy decisions and monitoring response to therapy in patients with ocular surface lesions. Its integration into routine practice offers a valuable, non-invasive supplement to slit-lamp biomicroscopy and histopathology.

## 1 | Introduction

Ocular surface lesions include a diverse group of pathologies involving the conjunctiva and cornea, ranging from benign conditions to aggressive malignancies. They include ocular surface squamous neoplasia (OSSN), pterygium and pinguecula, conjunctival melanoma (CM), primary acquired melanosis (PAM), conjunctival nevus, conjunctival lymphoma, benign reactive lymphoid hyperplasia (BRLH) and conjunctival amyloidosis. While some of these conditions like pterygium and pinguecula are primarily degenerative, others like CM have significant metastatic potential and require early detection and intervention. Differentiating between benign and cancerous lesions is critical since delayed diagnosis can lead to ocular and systemic consequences.

Imaging plays a valuable role in the evaluation of these lesions, supplementing clinical examination and histopathology. Traditional diagnostic methods rely on clinical acumen, visualisation with slit-lamp biomicroscopy and biopsy in certain cases. However, recent advances in imaging technology allow for non-invasive, high-resolution visualisation of ocular surface lesions to aid in early diagnosis, guide localisation of biopsy when indicated and as an adjunctive tool for monitoring response to treatment. Specifically, high-resolution anterior segment optical coherence tomography (AS-OCT) has gained prominence as an imaging modality for its ability to produce morphological cross sectional images of the anterior segment. It is a non-invasive approach that provides valuable insights into lesion depth, structural features and tissue layer involvement. As such, AS-OCT has the potential to guide clinicians in distinguishing between benign and malignant lesions and determining lesion subtype.

In this review, we discuss the clinical presentation and AS-OCT imaging features of ocular surface lesions including OSSN, pterygium and pinguecula, Salzman’s nodular degeneration, CM, PAM, conjunctival nevi, conjunctival lymphoma, BRLH and amyloidosis (Table 1). We explore how AS-OCT can be used to detect, characterise and monitor these lesions, representing a significant advancement in the management of ocular surface tumours.

## 2 | Principles of AS-OCT

AS-OCT is a non-invasive and contactless imaging modality that allows for cross sectional imaging of biological tissues [1]. AS-OCT utilises longer wavelengths (1000–1300 nm) compared to posterior segment OCT, allowing for better penetration of anterior segment structures [2–4]. Devices capable of 5–7 μm (high resolution) and less than 3 μm (ultra-high resolution) allow for excellent morphological details of ocular surface lesions [5, 6]. The non-contact nature and high resolution of AS-OCT make it valuable for assessing ocular surface lesions, aiding in diagnosis, treatment monitoring, and surgical planning.

## 3 | Interpretation of AS-OCT

The interpretation of AS-OCT relies on a systematic approach. First, one should locate normal epithelium. Normal epithelium is thin and appears as a dark, or hyporeflective, band compared to subepithelial tissue. In lesions involving the cornea and the conjunctiva, one should make the differentiation between the epithelium and sub-epithelium. When involving the cornea, subepithelial structures include Bowman’s layer and the anterior corneal stroma. It is important to note the reflectivity and thickness of the epithelium, along with any zones of transition from normal to abnormal epithelium. After the epithelium is identified and subepithelial tissue is characterised, the location of the lesion should be determined as either epithelial, subepithelial or both in some cases. This distinction is crucial, as it is useful in narrowing down the differential diagnosis. Once the location of the lesion is determined, it is important to note intralesional characteristics such as homogeneity, heterogeneity and reflectivity.

## 4 | AS-OCT Characteristics of Ocular Surface Lesions

### 4.1 | Ocular Surface Squamous Neoplasia

**FIGURE 1 |:**
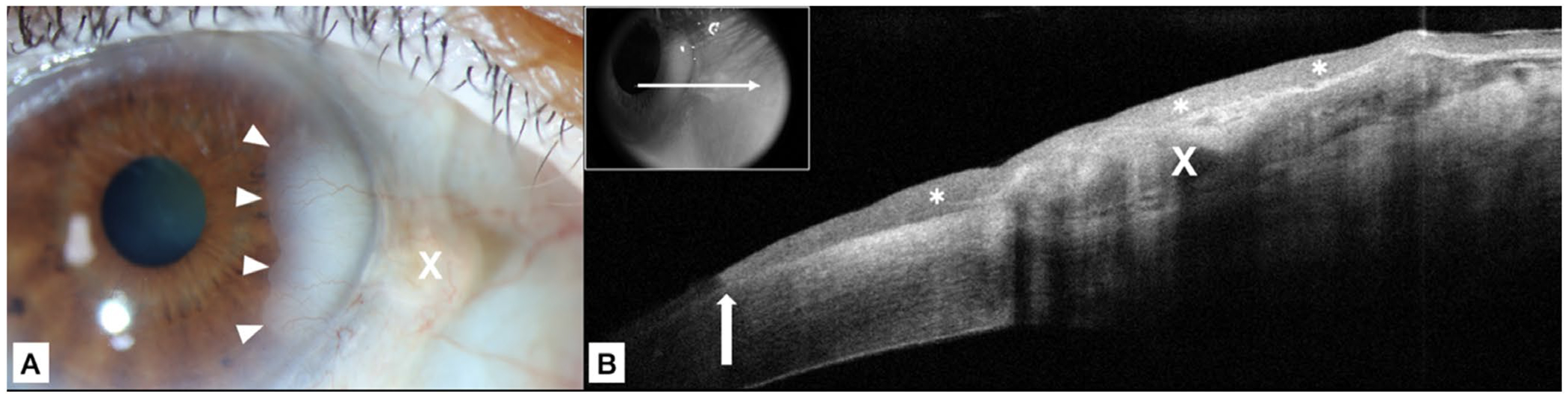
(A) An 87-year-old woman was referred for a lesion involving the cornea and conjunctiva of the right eye from 2 to 4 o’clock. Slit-lamp examination demonstrated an opalescent corneal lesion (arrowheads) with increased vascularity arising from an adjacent pinguecula (white X). Based on clinical suspicion and AS-OCT findings, treatment with 5-fluorouracil (5-FU) was initiated. (B) AS-OCT demonstrates a 90° sharply demarcated (white arrow), thickened and hyperreflective epithelium (white asterisks). Note the underlying pinguecula (white X).

**FIGURE 2 |:**
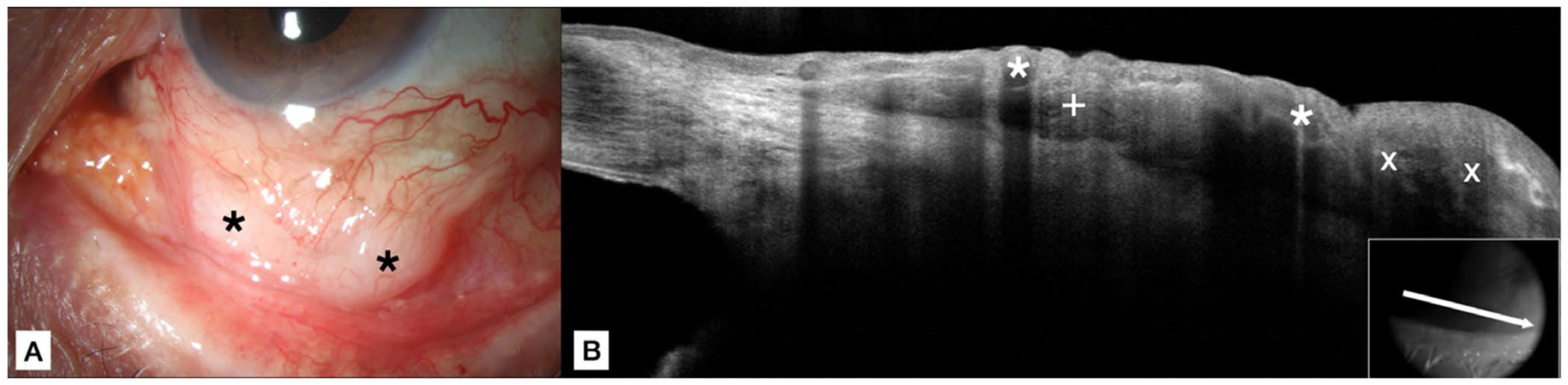
(A) An 89-year-old man was referred for a rapidly growing lesion in the left eye. Slit-lamp examination demonstrated an elevated subepithelial bulbar lesion from 4 to 9 o’clock with overlying feeder vessels (black asterisks). Biopsy confirmed invasive squamous cell carcinoma. (B) AS-OCT shows modest epithelial thickening (asterisk) with a prominent subepithelial lesion (white plus). Of note, there are areas where the epithelium penetrates into the subepithelial space (white X).

OSSN is a cancer of the ocular surface epithelium. It can range from minimal surface dysplasia to invasive squamous cell carcinoma [7, 8]. It occurs most often in White patients in the 6th to 7th decades of life, with an incidence of 3.4 per 100 000 patient-years, demonstrating almost a tripling from 2014 to 2021 in an IRIS Registry study [9]. Risk factors for OSSN include solar ultraviolet exposure, specific strains of human papilloma virus (HPV), human immunodeficiency virus (HIV) and cigarette smoking [10–14]. The incidence of OSSN in sub-Saharan Africa has been reported to be even higher, perhaps related to the higher rates of HIV/AIDS, HPV infection and sun exposure in these regions [11, 15]. Clinically, OSSN has variable appearances, presenting as a gelatinous, leukoplakic, opalescent, papillary and/or pigmented lesion (Figures 1A and 2A). Tumours may present as elevated lesions with feeder vessels, and when compared to benign lesions, OSSN is more likely to be papilliform [11]. OSSN tumours may also have irregular borders and a hairpin configuration of associated blood vessels [16, 17]. The gold standard for making a diagnosis of OSSN is with a biopsy and histopathologic analysis, which demonstrates thickened epithelium and full thickness faulty maturational sequencing. These morphologic changes are mirrored on AS-OCT [17–19]. Therefore, clinical features, physician acumen and characteristic features on AS-OCT highly suggestive of OSSN may allow ophthalmologists to initiate early treatment.

Historically, OSSN has been treated with surgical excision with wide margins. However, in recent years, topical chemotherapeutic eye drops have gained prominence and demonstrated efficacy, including 5-fluorouracil (5-FU), mitomycin C (MMC) and interferon alpha-2 beta (IFN). Surgical excision of lesions has risks, which include limbal stem cell deficiency in extensive excisions, dry eye, symblepharon and scarring [20]. Despite the surgeon’s best efforts, surgical margins may be positive, resulting in recurrence rates greater than 50%, and even those with negative margins can reach 33% [21]. The advantage of medical therapy is that it treats the entire ocular surface, addressing microscopic and subclinical disease. This is especially helpful in multifocal, annular, and recurrent lesions. In such cases, it is prudent to initiate medical therapy as the first-line treatment. Studies have demonstrated that 5-FU has similar efficacy to surgical excision. Furthermore, 5-FU is well-tolerated and achieves resolution in approximately 88% of treated tumours [22, 23]. MMC demonstrates similar efficacy to 5-FU and has a shorter treatment period, but has unpleasant short and long-term side effects including dry eye, punctal stenosis, limbal stem cell deficiency and persistent epithelial defects [24–27]. IFN has similar resolution rates to 5-FU and MMC, with one study reporting a complete response in 89% of patients treated with IFN and 92% of patients treated with MMC [27, 28]. IFN is generally well-tolerated with fewer side effects compared to MMC [29, 30]. As such, there has been a shift to increased use of topical monotherapies like 5-FU and IFN, and decreased use of MMC [31].

AS-OCT is a useful adjunctive diagnostic tool with over 90% specificity and sensitivity in making the diagnosis of OSSN [32–34]. A study by Garcia et al. obtained 100% sensitivity and specificity with a cutoff of 141 μm, and another study by Nanji and colleagues found similar metrics using a cutoff of 120 μm [35, 36]. The classic findings of OSSN on AS-OCT help to distinguish these malignant lesions from benign lesions, such as pterygia or Salzmann’s nodular degeneration.

On AS-OCT, OSSN demonstrates a 90° sharply demarcated, thickened and hyperreflective epithelium (Figure 1B). These characteristics parallel the histopathological changes [5]. When lesions are thick, posterior shadowing beneath the hyperreflective epithelium may be noted. AS-OCT also allows for monitoring of tumour response to topical therapies, and scans demonstrate a return to normal thickness, non-hyperreflective epithelium once the lesion has resolved. OCT scans demonstrating an angled or tapered transition zone are highly suggestive of pannus or metaplasia, not neoplasia [37].

AS-OCT may be helpful in distinguishing between non-invasive and invasive OSSN. In conjunctival intraepithelial neoplasia, AS-OCT shows a clear separation plane between the lesion and stroma, while invasive squamous cell carcinoma loses this plane and involves subepithelial extension (Figure 2B) [18]. Furthermore, Kaliki et al. described a ‘wedge sign’ on AS-OCT in invasive noduloulcerative OSSN corresponding to stromal tumour invasion [38]. The stromal wedge may represent the deep lateral spread of tumour cells beneath normal epithelium.

When obtaining OCT images of an OSSN, it is important to obtain multiple scans over and around the area of the lesion to capture any subtle findings. While OCT is highly specific and sensitive for making a diagnosis when the characteristic hyper-reflective epithelium and abrupt transition zone are present, patients with comorbid ocular pathologies (i.e., limbal stem cell deficiency, herpes keratitis) and those with small lesions may have images that are harder to interpret. In cases where AS-OCT findings are ambiguous, incisional or excisional biopsy is necessary.

### 4.2 | Pinguecula and Pterygia

**FIGURE 3 |:**
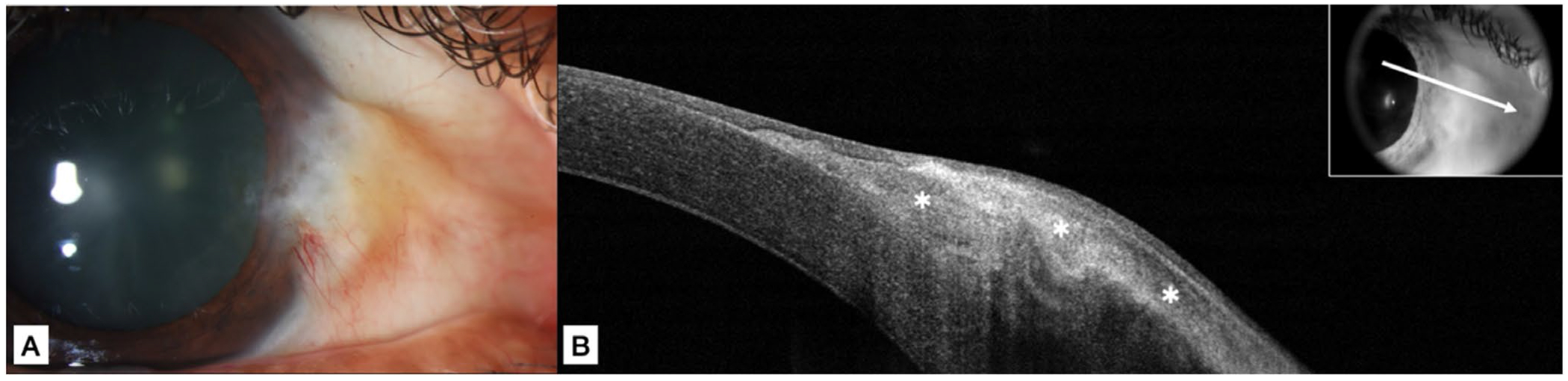
(A) A 75-year-old Hispanic male with an extensive history of sun exposure presented with a bothersome nasal conjunctival lesion in the right eye. Surgical excision was performed, and histopathology confirmed the diagnosis of pterygium. (B) AS-OCT reveals a subepithelial hyperreflective mass (white asterisks) extending onto the cornea with normal thickness epithelium.

Pinguecula and pterygia are benign degenerative lesions of the ocular surface associated with chronic UV and environmental irritant exposure. A pinguecula is a yellowish, slightly elevated conjunctival lesion that does not encroach upon the cornea. In contrast, a pterygium is a fibrovascular proliferation of conjunctival tissue that extends to the cornea (Figure 3A). Both lesions can cause irritation and redness, particularly in the case of pterygia and visual disturbance due to astigmatism or obscuration of the visual axis if more severe. These lesions are diagnosed by their characteristic clinical appearance at the slit-lamp biomicroscope, and management depends on symptoms, corneal involvement and cosmesis. Surgical excision of pterygia may be performed for patients with significant discomfort or visual issues.

On AS-OCT, pinguecula appear as a well-circumscribed subepithelial mass with variable reflectivity [39]. The epithelium is normal thickness and may be mildly hyperreflective. While pinguecula are easily identified on clinical exam, OSSN can emanate from these sun-exposed lesions, and the AS-OCT can help in this differentiation (Figure 1A,B).

AS-OCT of pterygia demonstrates a hyperreflective subepithelial mass that extends from the bulbar conjunctiva onto the cornea (Figure 3B). The epithelium can be of normal thickness or slightly thickened or thinned over the pterygium, with variable hyperreflectivity (most often slight hyperreflectivity) [40]. The subepithelial lesion is densely hyperreflective and fibrillary, having a string-like appearance that corresponds to the dense fibrovascular tissue making up a pterygium [41].

While pterygia are common lesions, they can sometimes be confounded clinically with OSSN, making the diagnosis somewhat complicated. Pterygium and OSSN have similar risk factors, occurring in patients with chronic UV exposure. Studies have shown that up to 15% of pterygium can harbour an occult OSSN [42–48]. If there is suspicion for an occult OSSN within a pterygium, it is important to note the presence of any abnormal blood vessels, opalescence or leukoplakia. Lesion location and epithelial thickness on AS-OCT can be helpful in differentiation, with pterygia appearing as a subepithelial lesion and OSSN presenting as a thickened epithelial lesion.

### 4.3 | Salzmann’s Nodular Degeneration

**FIGURE 4 |:**
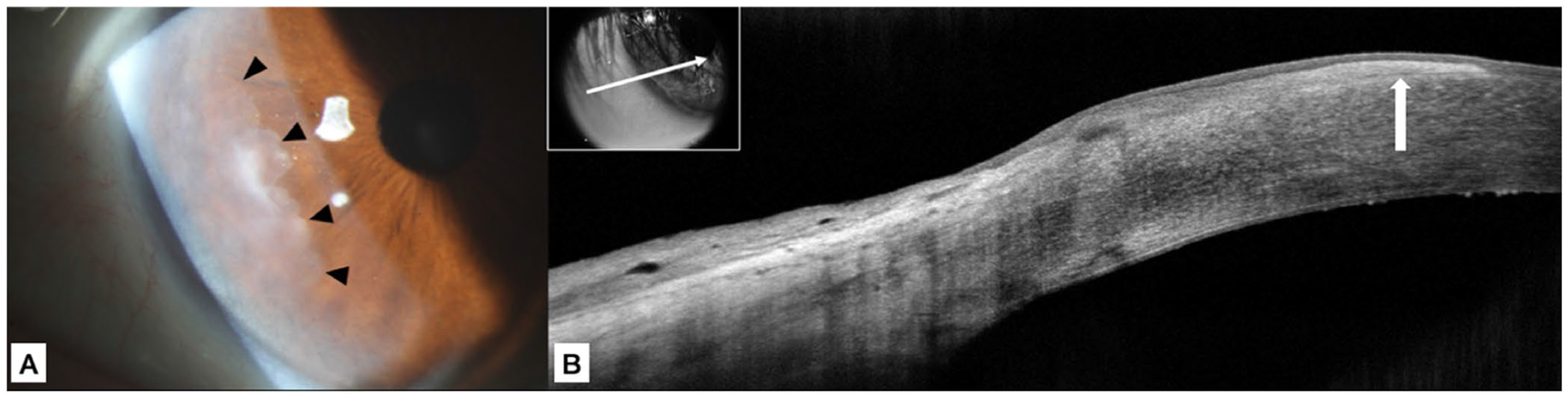
(A) A 74-year-old man with a history of extensive sun exposure and prior ocular surface squamous neoplasia in the left eye treated with 5-fluorouracil presented with a corneal lesion in the right eye, characterised by greyish nodules extending from 7:30 to 9 o’clock (black arrowheads). The lesion had been stable for several years, and clinical examination was suggestive of Salzmann’s nodular degeneration. (B) AS-OCT demonstrates a hyperreflective subepithelial lesion extending onto the cornea (white arrow) with normal overlying epithelium.

Salzmann’s nodular degeneration is a slowly progressive non-inflammatory condition characterised by whitish grey opacities usually located in the peripheral or mid-peripheral cornea (Figure 4A) [49]. Occasionally, Salzmann’s nodules can look like corneal OSSN, appearing as an opaque lesion at the limbus [50]. Management is determined by symptom severity. Asymptomatic lesions can be managed with observation and lubrication. In cases where the lesions are causing irregular astigmatism or irritation, surgical interventions like superficial keratectomy can be performed. Diagnosis is mainly clinical, and AS-OCT can be used to support the diagnosis.

AS-OCT of Salzmann’s nodules reveal hyperreflective subepithelial lesions wedged between the epithelium and Bowman’s layer (Figure 4B). AS-OCT is helpful to assess morphologic features and depth of nodules. Rarely, some nodules may show triangular spicules extending into the underlying tissue and stromal scarring beneath Bowman’s layer [51]. The central regions of the nodules display heterogeneous signal intensity, indicating variations in their internal density [52]. Nodules extending above Bowman’s layer may induce irregular astigmatism due to the elevation of the anterior corneal surface. The epithelium over the nodule is generally thin, helping to differentiate Salzmann’s nodules from OSSN [52].

### 4.4 | Conjunctival Melanoma

**FIGURE 5 |:**
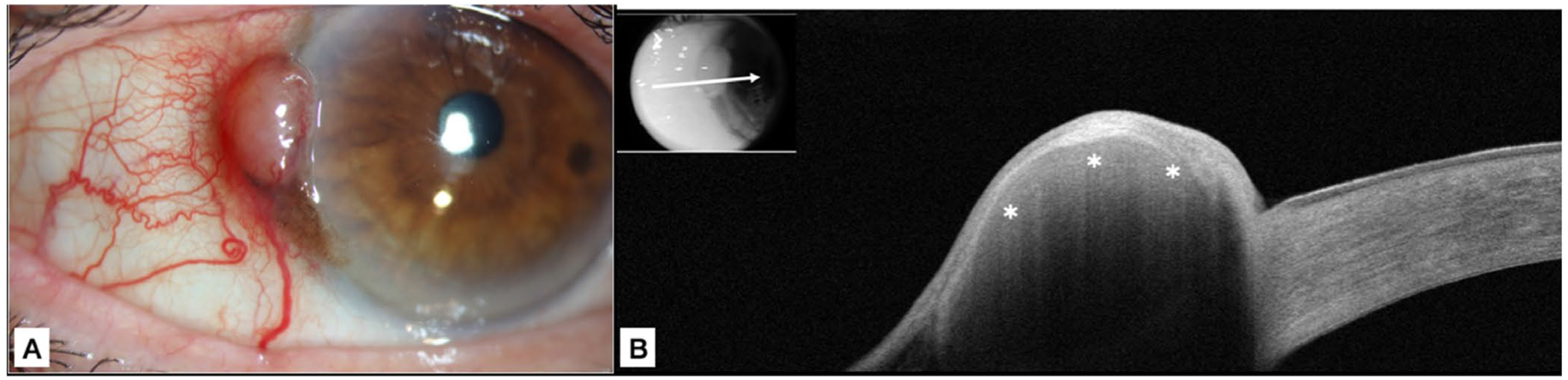
(A) A 74-year-old White male presented with a mixed-pigment elevated gelatinous lesion in the right eye extending from 8:30 to 9:30 o’clock, arising within an area of primary acquired melanosis spanning from 7:30 to 10 o’clock. The patient underwent surgical excision with cryotherapy to the margins, amniotic membrane transplantation, and plaque brachytherapy for positive deep margins. Histopathology confirmed the diagnosis of conjunctival melanoma. (B) AS-OCT shows a subepithelial hyperreflective lesion (white asterisks) with normal thickness epithelium. Note how posterior shadowing obscures the deeper half of the lesion.

CM can be a deadly disease with an incidence of 0.39 per million people, and it affects primarily White patients in the 7th and 8th decades of life [53]. Though it is rare, CM is the deadliest ocular surface tumour, with death occurring in 7% of patients by 5 years’ follow-up and 13% of patients by 8 years’ follow-up [54]. CM arises from melanocytes in the basal conjunctival epithelium. CM tumours present as a unilateral, heterogeneously pigmented lesion on the bulbar, limbal, forniceal and/or palpebral conjunctiva (Figure 5A) [55–57]. These lesions are highly vascularized, may be flat or nodular, and can increase in thickness over time. Occasionally, CM tumours may be amelanotic and contain minimal pigment.

CM most commonly arises from preexisting PAM with atypia (42%–74%), followed by de novo occurrence (11%–38%) and conjunctival nevus (2%–33%) [58].

The gold standard for diagnosing CM is by histopathologic assessment, in which atypical melanocytes are seen invading the substantia propria with loss of maturation and normal polarity [55]. Treatment of localised CM is with wide surgical excision using a ‘no-touch technique’ followed by cryotherapy to the margins [59–61]. Adjuvant therapies including topical chemotherapy and brachytherapy may be used depending on the case. Radiotherapy, targeted therapies including BRAF/MEK inhibitors and immune checkpoint inhibitors, and orbital exenteration may be necessary in more advanced disease [58, 62–68]. Molecular targeted therapy against BRAF/MEK in CM has demonstrated efficacy in patients with advanced disease [60, 69, 70]. There are several reports of unresectable or metastatic CM that have shown complete regression or disease stabilisation with the use of immune checkpoint inhibitors [71–74]. Orbital exenteration is a last-resort option when alternative eye-sparing therapies fail [75]. In cases of surgical excision with positive deep margins, plaque brachytherapy is the treatment of choice [76].

AS-OCT of CM demonstrates a hyperreflective subepithelial lesion (Figure 5B). The epithelium appears hyperreflective with variable thickening. Scans may also demonstrate regions of hyperreflective basal epithelium in areas of PAM [32]. Lesions that are highly pigmented can cause optical shadowing, making it more difficult to assess deeper margins, particularly in thick lesions [36, 77]. Amelanotic CM tumours can resemble OSSN; in these cases, AS-OCT is particularly helpful in distinguishing the two, as an amelanotic CM appears as a subepithelial hyper-reflective lesion while OSSN is a hyperreflective epithelial lesion [5, 78–80]. AS-OCT can also reliably differentiate between benign nevi and CM, as cysts are present in conjunctival nevi.

### 4.5 | Primary Acquired Melanosis

**FIGURE 6 |:**
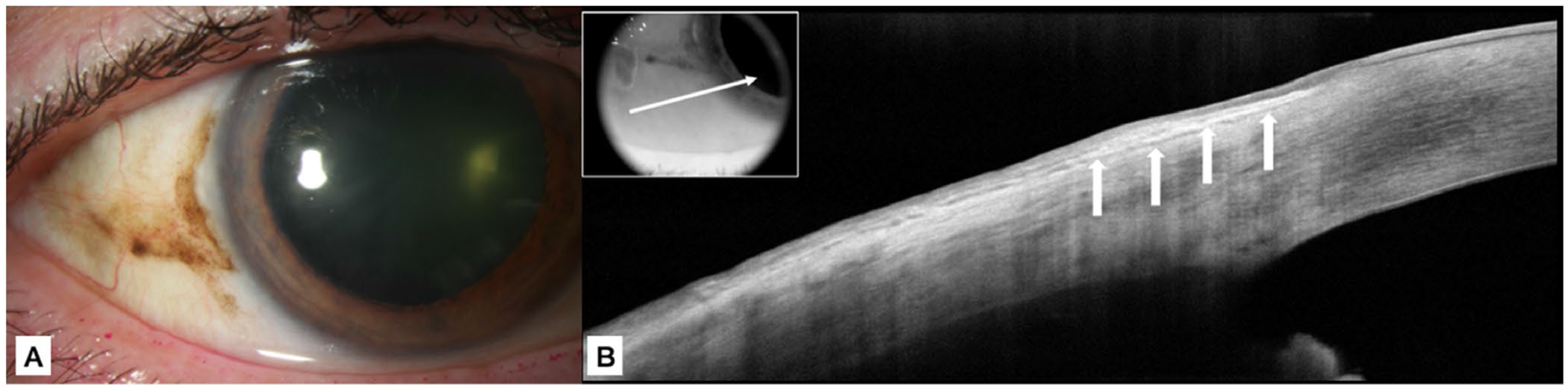
(A) A 76-year-old man was referred for evaluation of a new pigmented lesion on the bulbar conjunctiva of the right eye. The lesion was excised with wide margins and cryotherapy, and histopathology confirmed conjunctival melanosis with moderate atypia. (B) AS-OCT reveals an epithelial lesion with hyperreflectivity of the basal epithelium (white arrows) and without extension to the subepithelial space.

PAM with atypia is a significant precursor to CM, with up to 46% risk of malignant transformation for lesions with severe atypia [81, 82]. PAM is a unilateral, flat pigmented lesion arising from intraepithelial melanocytes. It is non-cystic, patchy or diffuse and irregularly pigmented [83]. It is usually seen in White, middle-aged adults; the precise population incidence remains unknown; however, one study shows that PAM was present in 36% of European Whites [84]. Clinically, PAM presents as irregularly bordered pigmentation involving the bulbar, limbal, and less commonly forniceal and palpebral conjunctiva (Figure 6A) [81]. The gold standard for diagnosis is incisional or excisional biopsy followed by histopathologic grading [85]. Lesions with atypia are treated with wide excision, adjunctive cryotherapy, topical immunotherapy with interferon or topical chemotherapy such as MMC [86]. Patients with CM tumours that arise from PAM tend to present with greater clock hour involvement; thus, more extensive PAM lesions should be promptly excised [87]. The risk of progression from PAM to melanoma increases by 1.7-fold for each additional clock hour of conjunctival involvement [88]. Clinical observation is recommended for lesions with no atypia.

AS-OCT of PAM demonstrates a flat epithelial lesion characterised by basal epithelial hyperreflectivity and no subepithelial extension (Figure 6B) [85]. The hyperreflective basal epithelium on AS-OCT correlates histologically to the basal epithelial melanocytic pigmentation [5]. On OCT, the degree of optical shadowing corresponds to the level of melanocytic pigmentation seen in the histopathologic specimen [85].

It is important to note that OCT cannot differentiate the degree of atypia in PAM lesions, which is essential for assessing malignant potential [5, 85]. In addition, OCT cannot differentiate PAM from complexion-associated melanosis (CAM), which is a benign and usually bilateral conjunctival pigmentation occurring in patients with darker skin complexion [39]. Making the distinction between PAM and CAM requires knowledge of the entire clinical picture and patient history. Although AS-OCT cannot determine cytologic atypia in PAM, it may assist in the initial assessment of a suspicious conjunctival pigmented lesion to check for the presence of subepithelial involvement, which would be more suggestive of melanoma. Histopathologic analysis is essential to determine the level of atypia in PAM and guide further management.

### 4.6 | Conjunctival Nevus

**FIGURE 7 |:**
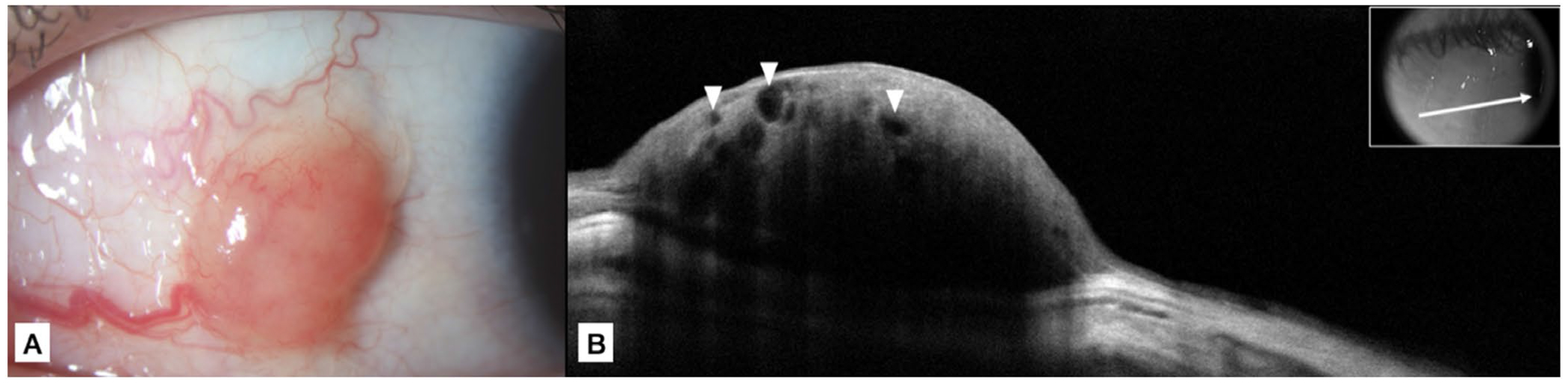
(A) A 9-year-old girl was referred by her ophthalmologist for evaluation of a conjunctival lesion suspicious for ocular surface squamous neoplasia. On presentation, she had an amelanotic, elevated, mobile lesion with a temporal feeder vessel in the left eye. (B) AS-OCT shows a well-demarcated subepithelial lesion with homogeneous internal reflectivity, smooth margins and intralesional cysts (white arrowheads). Clinical examination and serial AS-OCT were consistent with a conjunctival nevus, and she was monitored annually. Six years later, the lesion was excised for cosmetic reasons, and histopathology confirmed a compound cystic nevus.

Conjunctival nevi are the most common melanocytic lesion of the conjunctiva, accounting for over 50% of pigmented conjunctival lesions [89]. Conjunctival nevi are benign melanocytic proliferations arising from nests of nevus cells typically located in the subepithelial stroma [90, 91]. These lesions are usually unilateral, well-circumscribed, slightly elevated and may be pigmented or amelanotic (Figure 7A). They are most often located on the interpalpebral bulbar conjunctiva, commonly near the limbus and pigmentation is present in approximately 84% of cases. Conjunctival nevi are most frequently found in White individuals with a mean age of 32 [92]. Diagnosis is primarily clinical, with excisional biopsy reserved for lesions demonstrating rapid growth, atypical vascularity or irregular pigmentation or location [93]. Nevi can be categorised into three main histopathologic subtypes based on melanocyte location: junctional, compound and subepithelial [90]. Nevi often contain cysts, which can be seen on histopathology and correlate to the AS-OCT findings [18]. Management is typically observational, with excision considered for cosmetic reasons or suspicion of malignant transformation, although this is rare (< 1%) [92].

On AS-OCT, conjunctival nevi appear as well-demarcated subepithelial lesions with homogeneous internal reflectivity and smooth margins [36, 91]. A distinguishing hallmark is the presence of multiple hyporeflective intralesional cysts (Figure 7B), seen in the majority of cases and indicative of lesion chronicity and benignity [94]. The overlying epithelium is typically normal. Posterior shadowing may occur in heavily pigmented lesions, though overall lesion architecture is usually well-visualised [91].

AS-OCT enhances diagnostic confidence by confirming the characteristic cystic and subepithelial features of conjunctival nevi. This is particularly valuable when deciding to observe rather than biopsy stable lesions with benign morphology. In rare instances of malignant transformation, serial OCT imaging can aid in early detection by revealing structural changes such as new mass growth. However, OCT has some limitations in the evaluation of conjunctival nevi. Heavily pigmented lesions may cause posterior shadowing, limiting visibility of lesion depth or subtle changes over time [91]. Additionally, while intralesional cysts strongly support a benign diagnosis, OCT cannot assess cytologic atypia and biopsy is recommended in atypical or evolving lesions.

### 4.7 | Conjunctival Lymphoma

Conjunctival lymphoma is a rare presentation of extranodal lymphomas, comprising only 5%–10% of all extranodal lymphomas [95]. It arises from lymphoid tissue in the subepithelial stroma of the conjunctiva and typically presents in older adults, most often between the sixth and seventh decades of life.

**FIGURE 8 |:**
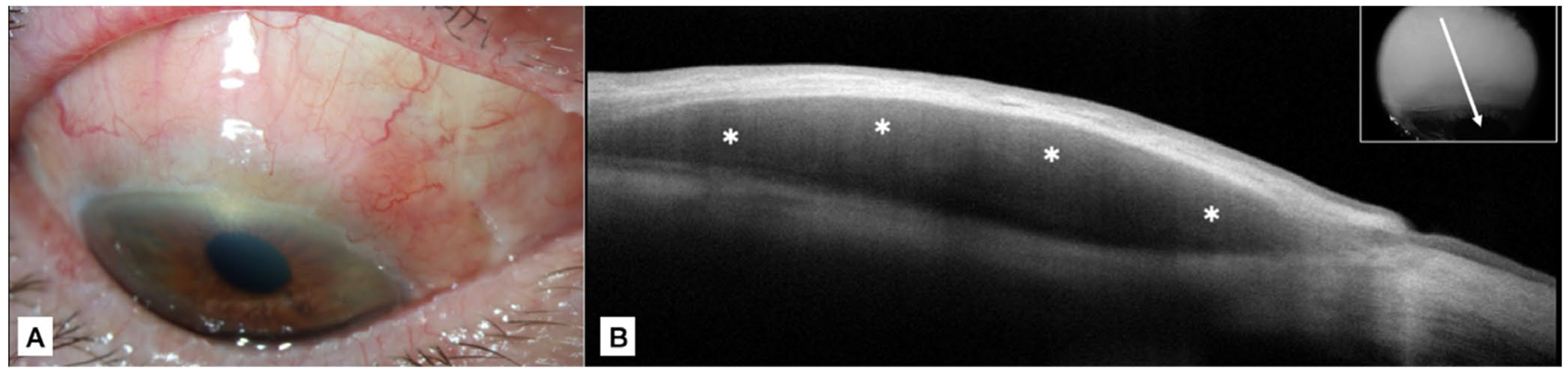
(A) A 75-year-old man was referred for evaluation of a superior salmon-patch lesion in the right eye. He had previously been diagnosed with superior limbic keratoconjunctivitis. AS-OCT revealed features suggestive of a lymphoproliferative process, prompting biopsy. Histopathology confirmed marginal zone lymphoma. The patient was treated with ultra–low-dose radiotherapy and has remained recurrence-free to date. (B) AS-OCT demonstrates a subepithelial hyporeflective mass with homogenous internal reflectivity. The lesion is well-demarcated with a monomorphic dot-like granularity (white asterisks).

Conjunctival lymphoma lesions appear as a painless, smooth, salmon-coloured patch on the conjunctiva (Figure 8A) or as chronic follicles [96]. Bilateral involvement occurs in approximately 10%–15% of cases. Conjunctival lymphoma may occur as a primary ocular tumour or be associated with systemic lymphoma, which is present in up to 10%–32% of cases at diagnosis [97].

Definitive diagnosis requires an incisional biopsy. Histopathology demonstrates a dense monomorphic lymphoid infiltrate, which morphologically correlates with the AS-OCT findings [18, 98]. Immunohistochemistry reveals markers such as CD20+ and BCL2+ [95, 99]. Flow cytometry or gene-rearrangement studies are used to confirm B-cell clonality. Systemic workup, including positron emission tomography (PET-CT) and possibly bone marrow biopsy, is recommended to rule out disseminated disease [95]. Treatment varies by extent of disease and may include radiotherapy or systemic chemotherapy. Ultra-low–dose radiotherapy with 4 Grey delivered in two fractions has been shown to be an effective treatment for localised disease [99–101]. Prognosis is generally favourable in localised cases [96, 97, 99, 100].

AS-OCT of conjunctival lymphoma shows a hyporeflective subepithelial lesion with a homogeneous internal reflectivity and dot-like internal granularity (Figure 8B). The lesion has smooth and regular anterior borders, and the overlying epithelium is intact and not thickened. A thin hyperreflective anterior surface line may be observed surrounding the lesion, corresponding to compression of the substantia propria by the underlying lesion [98].

AS-OCT serves as a helpful adjunct in assessing conjunctival lymphoma. Although often clinically obvious, conjunctival lymphoma can masquerade as chronic episcleritis, pingueculitis, nodular scleritis, superior limbic keratoconjunctivitis or other ‘red eye’ conditions. OCT is not routinely used to document scleritis, which is a clinical diagnosis, but scans of scleritis demonstrate an increase in hyporeflective spaces which correspond to dilated superficial and deep episcleral blood vessels [102]. In cases of suspected ‘red eye’ conditions that fail to respond to standard anti-inflammatory treatment, a masquerade should be considered. AS-OCT can help reveal the possible presence of a lymphoproliferative process and guide the decision to proceed with biopsy. It allows for detailed evaluation of lesion thickness, lateral extent and surface contour, which can assist in targeting biopsy sites, especially when the lesion is subtle or diffusely spread. OCT can also be used for longitudinal monitoring, particularly to evaluate treatment response following radiotherapy, where progressive thinning or resolution of the hyporeflective lesion can be observed [96]. While AS-OCT can be helpful to differentiate lymphoma from other lesions such as conjunctival amyloidosis based on its uniform subepithelial profile, it cannot reliably distinguish malignant lymphoma from BRLH, nor can it confirm clonality [96]. Thus, histopathologic analysis remains essential for definitive diagnosis and classification.

### 4.8 | Benign Reactive Lymphoid Hyperplasia

**FIGURE 9 |:**
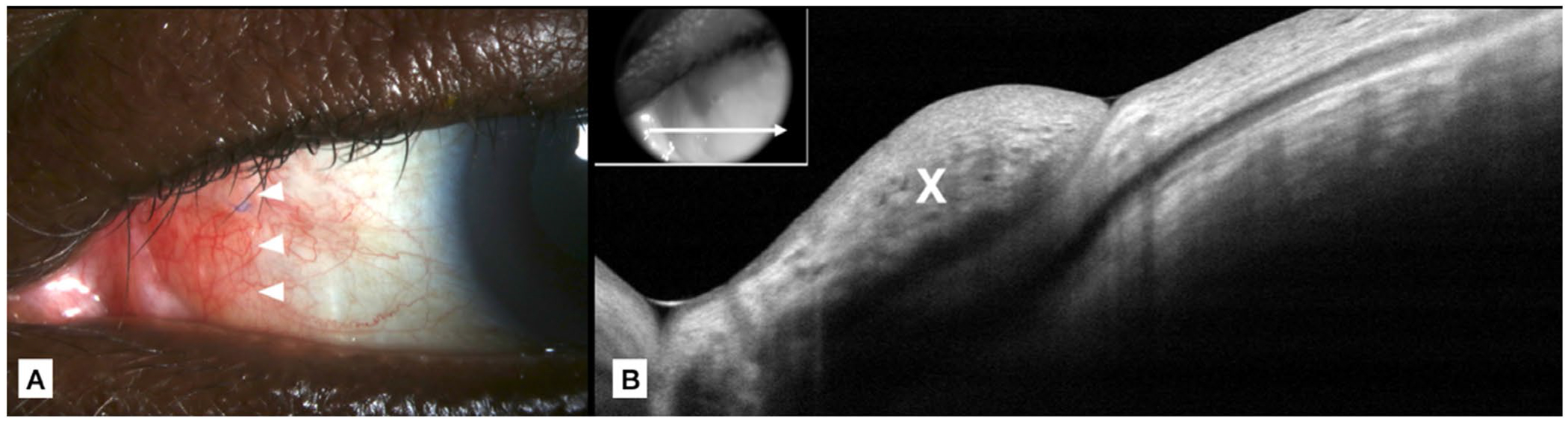
(A) A 65-year-old woman was referred for a fleshy, salmon-coloured lesion (arrowheads) near the plica of the left eye that was concerning for lymphoma. A biopsy was performed, and histopathology was negative for lymphoma and confirmed benign reactive lymphoid hyperplasia. (B) AS-OCT demonstrates a heterogeneous subepithelial lesion (white X). In this case, the heterogenous appearance of the lesion on AS-OCT was helpful in ruling out lymphoma and suggesting the diagnosis of benign reactive lymphoid hyperplasia.

BRLH of the conjunctiva is a benign proliferation of lymphoid tissue. BRLH is indistinguishable from conjunctival lymphoma on clinical examination, appearing as a salmon-patch lesion on the bulbar conjunctiva or fornix (Figures 8A and 9A) [103]. BRLH comprises 23% of ocular adnexal lymphoid tumours and usually occurs in older children and young adults [104, 105]. BRLH generally occurs in younger patients compared to lymphoma and is not associated with systemic disease.

**FIGURE 10 |:**
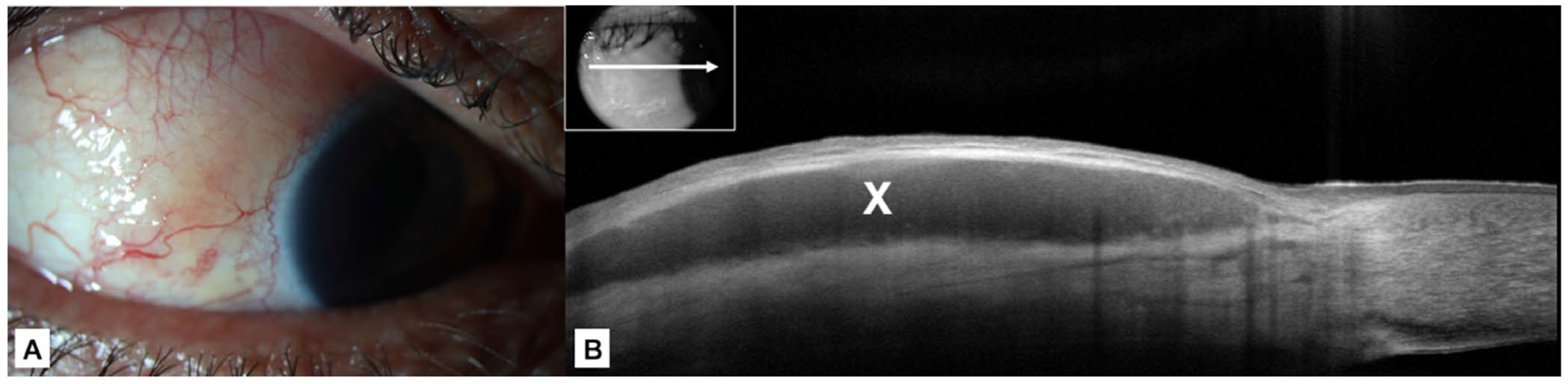
(A) A 46-year-old man was referred for bilateral salmon-patch lesions on the bulbar conjunctiva, which had shown partial response to topical steroids. (B) AS-OCT demonstrates a hyporeflective subepithelial infiltrate (white X) that appears very similarly to conjunctival lymphoma with overlying epithelium of normal thickness. Due to concern for lymphoma, a biopsy was performed which confirmed the diagnosis of benign reactive lymphoid hyperplasia (BRLH). In this case, AS-OCT was unable to reliably distinguish BRLH from lymphoma.

On AS-OCT, BRLH presents as a subepithelial infiltrate which may appear heterogenous or monomorphic by OCT (Figures 9B and 10B) [98]. The epithelium is normal. These lesions may appear as a mixed infiltrate that is clearly distinguishable from conjunctival lymphoma (Figure 9B) or may closely resemble conjunctival lymphoma (Figure 10B). The variable character of BRLH depends on the level of cellular infiltration. When BRLH looks similar to conjunctival lymphoma (homogenous lesion containing dot-like infiltrates), histopathology demonstrates a hypercellular infiltration in the subepithelial tissue [98]. On the other hand, hyperreflective BRLH lesions correlate to a paucicellular infiltrate on histology.

Although BRLH can appear clinically indistinguishable from conjunctival lymphoma and conjunctival amyloidosis, OCT may provide valuable clues. A heterogeneous infiltrate favours BRLH, whereas a homogeneous, well-demarcated lesion is more characteristic of lymphoma. OCT can also help differentiate BRLH from amyloidosis, which often shows hyperreflective linear infiltrates, and from inflammatory lesions, which typically appear irregular and ill-defined. When the OCT demonstrates a homogeneous infiltrate, a biopsy is warranted to rule out lymphoma. Ultimately, histopathology, cytology and gene-rearrangement studies are necessary to make an accurate distinction between benign and malignant lymphoproliferative diseases.

### 4.9 | Amyloidosis

**FIGURE 11 |:**
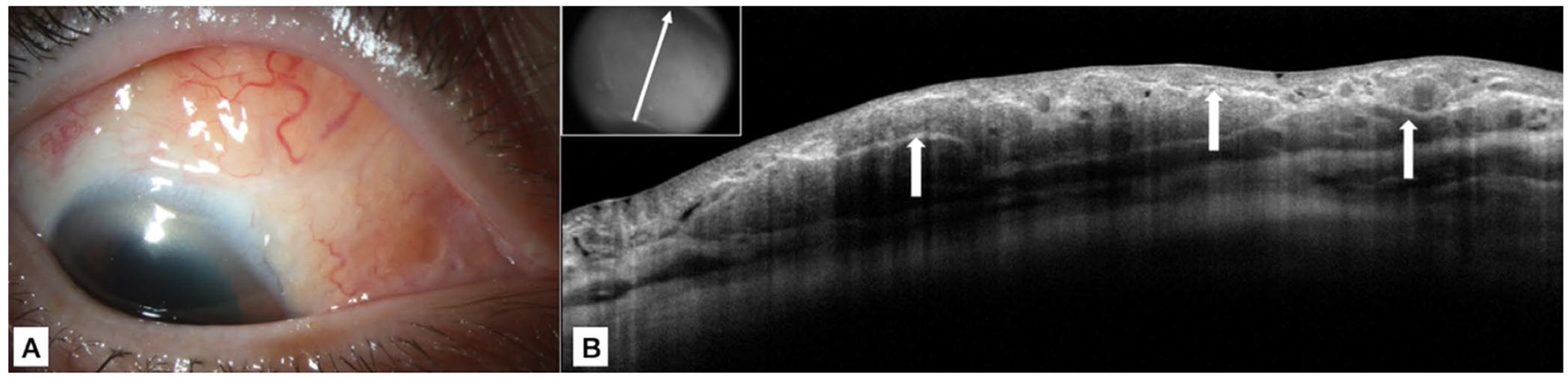
(A) A 71-year-old woman was referred for a superior, yellowish, fleshy lesion on the bulbar conjunctiva of the right eye. Her medical history was significant for systemic amyloidosis with documented lacrimal gland involvement. (B) AS-OCT shows a heterogeneous subepithelial lesion with hyperreflective linear infiltrates (white arrows). A biopsy of the lacrimal gland revealed amyloidosis. Management of the ocular surface manifestation was deferred, as she was already undergoing treatment for her systemic disease.

Conjunctival amyloidosis is a rare condition characterised by localised deposition of amyloid protein in the conjunctiva. Conjunctival amyloidosis can appear as a chronic conjunctivitis, chemosis or similar to conjunctival lymphoma, leading to diagnostic delay. It may occur idiopathically or as part of systemic amyloidosis. Amyloidosis refers to a group of diseases characterised by the abnormal deposition of amyloid fibrils in tissues and organs, with more than 30 distinct types identified. These proteins can deposit in several organs, including the kidney, heart, nervous system and eye. While the exact incidence of conjunctival involvement is unknown, in one study with 2455 conjunctival lesion pathology reports, only five patients were found to have amyloidosis [106]. Clinically, amyloid presents as a painless, yellowish-pink, red or pale lesion that appears irregular, thickened or multilobulated, and is typically located on the bulbar or tarsal conjunctiva (Figure 11A) [107].

Diagnosis is made by biopsy demonstrating eosinophilic, acellular, amorphous deposits, with Congo red staining confirming amyloid through apple-green birefringence under polarised light [106]. This heterogeneous histopathological morphology is reflected on the AS-OCT [98]. A workup includes serum and urine protein electrophoresis. In some cases, an abdominal fat pad biopsy can confirm systemic involvement [108]. Treatment of amyloidosis is complex due to the variety of etiologies. Management of conjunctival amyloidosis consists of treating underlying chronic inflammatory diseases; chemotherapy, steroids or stem cell transplantation for plasma cell dyscrasias; or surgical debulking with or without cryotherapy for localised cases [108].

AS-OCT can be particularly helpful in masquerade cases by revealing an infiltrating depositional lesion. In conjunctival amyloidosis, OCT demonstrates a heterogeneous, subepithelial lesion with hyperreflective linear infiltrates, poorly defined margins, and posterior shadowing in areas of denser amyloid deposition (Figure 11B) [40, 98]. The overlying epithelium may be mildly distorted or displaced.

The irregular, hyperreflective and heterogeneous appearance of amyloid deposits on OCT contrasts with the smooth, hyporeflective and homogeneous profile of conjunctival lymphoma and the inflammatory thickening of scleritis. These differences in internal architecture and contour help support clinical differentiation between lesions that appear similar on physical exam. Recognising the OCT morphology not only aids in differentiating amyloidosis from other lesions but also places this disease entity on the clinician’s differential diagnosis, ensuring that biopsy with Congo red staining/apple-green birefringence is performed for confirmation. AS-OCT helps characterise the depth and contour of subepithelial deposits in conjunctival amyloidosis and is particularly useful for targeting biopsies in irregular or multilobulated lesions. AS-OCT is also helpful for documenting progression or recurrence during follow-up.

## 5 | Limitations of AS-OCT

AS-OCT is a valuable, non-invasive tool for characterising ocular surface lesions; however, several limitations must be acknowledged. Thick or pigmented tumours often cause shadowing which can obscure the posterior margins. This may occur in cases of CM, thick OSSN or heavily keratinized lesions [36, 109]. In addition, AS-OCT lacks the cellular-level resolution needed to assess the degree of cytologic atypia, thus it cannot distinguish between PAM and CAM [36, 110]. Furthermore, AS-OCT of inflammatory ocular surface conditions may show epithelial hyperreflectivity, thereby making it difficult to interpret. False negatives may occur if the area of interest is not scanned. Ophthalmic imagers may need explicit guidance to ensure that the pathology of interest is appropriately captured.

In cases where the utility of AS-OCT is limited, other imaging modalities such as in vivo confocal microscopy (IVCM) and ultrasound biomicroscopy (UBM) play complementary roles in the evaluation of ocular surface tumours. IVCM has the advantage of providing cellular-level detail with a resolution of 1–2 μm, which can visualise nuclear atypia. However, IVCM is operator-dependent, often contact-based, provides a limited field of view, and requires expertise for interpretation [111]. As mentioned above, shadowing may occur in thicker and pigmented lesions on AS-OCT, and UBM has the advantage of providing deeper penetration that is useful for assessing tumour extension into the sclera or ciliary body. However, the resolution of UBM is 25–50 μm and will not provide the level of detail seen with other imaging modalities [112–114]. Understanding the relative strengths and limitations of each imaging modality allows for tailored and complementary imaging based on clinical suspicion and lesion characteristics.

## 6 | Conclusion

Ocular surface lesions encompass a wide spectrum of disorders ranging from benign degenerative changes to potentially life-threatening malignancies. Early and accurate differentiation among these lesions is crucial to avoid delays in treatment. AS-OCT is a valuable non-invasive imaging modality that complements clinical and histopathologic evaluation. In clinical practice, AS-OCT serves as an adjunct, providing additional diagnostic guidance particularly in challenging or ambiguous conjunctival tumours. It is helpful in distinguishing benign from malignant lesions and when monitoring treatment response or recurrence. By providing detailed cross sectional visualisation of epithelial and subepithelial structures, AS-OCT allows for early detection and effective monitoring of ocular surface tumours.

## Figures and Tables

**TABLE 1 |:** Summary of AS-OCT findings of ocular surface lesions.

Lesion	Location	Epithelial thickness	Lesion reflectivity	HR-OCT appearance
Non-invasive ocular surface squamous neoplasia (OSSN)	Epithelial	Thickened	Hyperreflective epithelium	Thickened hyperreflective epithelium with an abrupt 90° transition from normal to abnormal epithelium
Invasive OSSN	Epithelial and subepithelial	Slightly ↑/normal	Hyperreflective epithelium	Subepithelial hyperreflectivity with shadowing; the overlying epithelium is hyperreflective with variable thickness
Pinguecula	Subepithelial	Normal	Variable	Well-circumscribed subepithelial elevation
Pterygium	Subepithelial	Normal/slightly ↑ or ↓	Hyperreflective	Fibrillary, ‘stringy’ subepithelial mass extending from the bulbar conjunctiva onto the cornea
Salzmann’s nodular degeneration	Subepithelial (between the epithelium and Bowman’s layer)	Thinned over nodule	Hyperreflective	Hyperreflective subepithelial nodule with central regions displaying heterogeneous signal intensity
Conjunctival melanoma	Subepithelial ± basal epithelial changes	Normal/mildly ↑	Hyperreflective	Hyperreflective subepithelial mass; thick or highly pigmented lesions may cause posterior shadowing
Primary acquired melanosis (PAM)	Epithelial (starts at the basal layer)	Normal/mildly ↑	Hyperreflective	Flat lesion with basal epithelial hyperreflectivity without subepithelial extension
Conjunctival nevus	Subepithelial	Normal	Homogeneous with hyporeflective cysts	Well-demarcated subepithelial mass with multiple intralesional hyporeflective cysts
Conjunctival lymphoma	Subepithelial	Normal	Hyporeflective	Well-circumscribed, hyporeflective homogeneous mass with monomorphic dot-like internal granularity; thin hyperreflective anterior line surrounding the lesion may be present and corresponds to compression of the substantia propria
Benign reactive lymphoid hyperplasia (BRLH)	Subepithelial	Normal	Variable	Subepithelial infiltrate with heterogeneous internal pattern; homogeneous lesions can mimic lymphoma
Conjunctival amyloidosis	Subepithelial	Normal	Mixed reflectivity with hyperreflective linear foci	Heterogeneous subepithelial lesion with hyperreflective ribbon-like deposits and irregular margins

## Data Availability

Data sharing not applicable to this article as no datasets were generated or analysed during the current study.
